# Low Susceptibility of Pigs against Experimental Infection with HPAI Virus H5N1 Clade 2.3.4.4b

**DOI:** 10.3201/eid2907.230296

**Published:** 2023-07

**Authors:** Annika Graaf, Ronja Piesche, Julia Sehl-Ewert, Christian Grund, Anne Pohlmann, Martin Beer, Timm Harder

**Affiliations:** Friedrich-Loeffler-Institut, Greifswald–Insel Riems, Germany

**Keywords:** pigs, HPAIV, H5N1, zoonosis, mammal, panzootic, goose/Guangdong

## Abstract

We found that nasal and alimentary experimental exposure of pigs to highly pathogenic avian influenza virus H5N1 clade 2.3.4.4b was associated with marginal viral replication, without inducing any clinical manifestation or pathological changes. Only 1 of 8 pigs seroconverted, pointing to high resistance of pigs to clade 2.3.4.4b infection.

Spread of highly pathogenic avian influenza (HPAI) virus H5N1 clade 2.3.4.4b of the goose/Guangdong (gs/GD) lineage, has exacerbated since early 2022 into a panzootic (*1*). Regional enzootic status in wild bird populations in Europe and North America, with lethal courses of HPAI virus infection in some species, produced large numbers of wild bird carcasses, easy prey for raptors and scavengers. Exposure of terrestrial carnivores and marine mammals resulted in sporadic infections, some of those terminating with fatal encephalitis (*2*). Frequent spill-over events, rather than consistent mammal-to-mammal transmission, were at the basis of these cases (Figure, panel A). However, recently reported HPAI outbreaks among sea lions along the Pacific coast of South America and an outbreak in a mink farm in Spain (*3*) may constitute first examples of avian-independent transmission chains and increase public health concerns about zoonotic transmissions of this virus. Still, the total of 11 human cases globally reported for the currently dominating H5N1 2.3.4.4b lineage did not point toward increased zoonotic propensity (*4*).

Possible adaption of avian influenza virus (AIV) to mammalian livestock hosts and subsequent human exposure is of particular concern. In this respect, the role of pigs as a “mixing vessel” for HPAI viruses is largely unresolved. AIV can potentially be transmitted to pigs, and further reassortment with swine influenza A viruses (swIAVs) may contribute to the emergence of pandemic strains. Rare and subclinical infections of pigs with gs/GD HPAI virus have been confirmed serologically in Vietnam, Thailand, and France (*5*) and virologically in Indonesia (clades 2.1.1 and 2.1.3), Nigeria (clade 2.3.2.1c), China (clade 2.3.4), and Italy (clade 2.3.4.4.b) (*6*–*8*).

For our study, we purchased 4-month-old pigs (6 male, 4 female) from a conventional pig holding in Germany and exposed them nasally or by the alimentary route to high doses of the recent avian-derived HPAI virus H5N1 2.3.4.4b isolate A/chicken/Germany/AI04286/2022 (genotype Ger-10.21-N1.5). The egg-grown isolate was closely related to a mammal case but lacked any mammalian-adaptive mutations (Figure, panel A). We inoculated 2 groups of 4 pigs each intranasally or by feeding 1 infected embryonated chicken egg per animal. One sentinel pig per group was associated at day 1 postinoculation to assess the transmission by direct contact to those inoculated (Figure, panel B).

**Figure:**
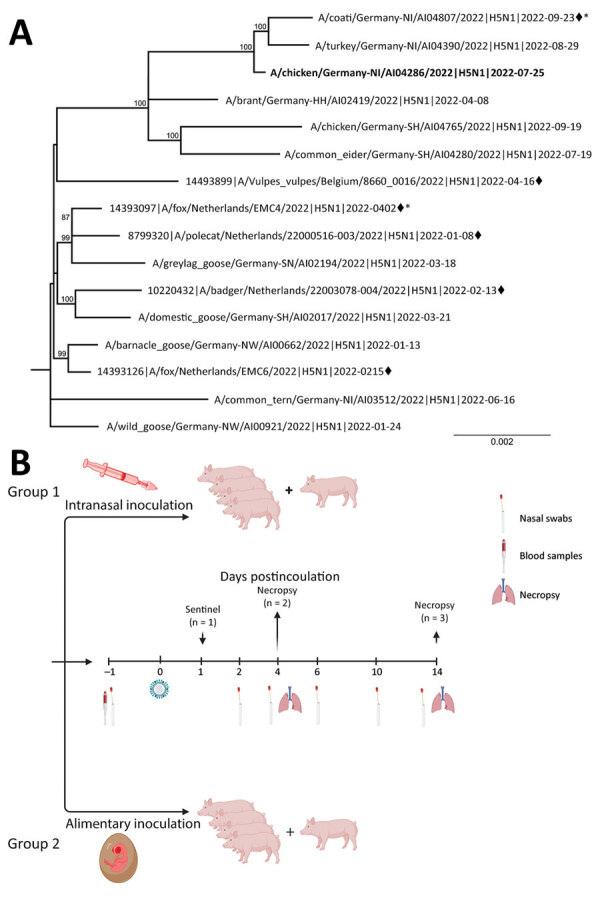
Phylogeny and experimental design for study of susceptibility of pigs against experimental infection with highly pathogenic avian influenza (HPAI) (H5N1) virus clade 2.3.4.4b. A) Maximum-likelihood phylogenetic tree (RAxML, https://cme.h-its.org/exelixis/web/software/raxml) based on 8 concatenated genome segments of selected recent HPAI H5N1 viruses from naturally infected avian hosts and from mammalian hosts (black diamonds) in Europe. Bold indicates study isolate A/chicken/Germany/AI04286/2022. Asterisks (*) indicate sequences with polymerase basic 2 E627K mutations. B) Scheme of the experimental setting of HPAI H5N1 virus infection of pigs. Four pigs, 4 months of age, were inoculated with 10^6^ TCID_50_ in 2 mL using mucosal atomization devices. Four pigs were each fed with 1 HPAI H5N1 virus–infected embryonated chicken egg, carrying ≈10^8^–10^9^ TCID_50_/mL of allantoic fluid. Each pig was offered an egg, separately, in a trough and observed to complete consume it. Ten-day-old eggs were inoculated with 0.2 mL of clarified amnio-allantoic fluid of egg passage 1 and incubated for 3 days or until embryonic death was evident. Eggs were chilled until fed to pigs. Panel B created with BioRender.com and licensed by the company (agreement no. UC258UM8J3). TCID_50_, 50% tissue culture infectious dose.

After exposure, HPAI virus H5N1 detection by real-time RT-PCR was limited to day 2 (intranasal group) and day 4 (oral group) postinoculation, with a range of 10–150 genome copy equivalents per 0.1 mL (Table). An avian-derived, swine-adapted H1avN1 strain intranasally inoculated into naïve pigs at the same dose and by the same device produced 3–4 log_10_ levels greater nasal excretion in comparison (Table) (*9*). It cannot be excluded that HPAI virus H5N1 detected in nasal swabs at day 2 postinoculation still represents residual inoculum. Correspondingly, except for 1 alimentary inoculated that showed tracheal viral loads at day 4 postinoculation close to the detection limit, samples from the remaining 3 pigs euthanized at day 4 postinoculation gave no indication of viral replication in respiratory or gut tissues, regardless of the inoculation route. Only in 1 intranasally inoculated animal, euthanized at day 14 postinoculation, was viral RNA detected at low levels in organ samples. In addition to samples from the respiratory and alimentary tracts, minute amounts of RNA were found also in a brain sample of that pig. The virus could not be isolated using chicken hepatoma cells and MDCK-II cells, and histologic and immunohistochemical investigations gave no evidence for inflammatory reactions or presence of viral antigen. Low viral loads in this pig impeded sequence analysis of eventual mutants. Nonetheless, this pig was the only animal that seroconverted at 14 dpi. In agreement with the virologic findings, none of the pigs showed any clinical signs or fever within 14 days of observation.

**Table:** Detection of seroconversion and of influenza A viral loads in tissues and nasal swabs of experimentally infected pigs exposed by intranasal or alimentary inoculation with HPAI virus H5N1 clade 2.3.4.4b virus*

Infection route	Day postinoculation	Animal ID	GEq, by qRT-PCR							NP-ELISA Seroconversion
			Nasal swabs†	Conchae nasalis	Trachea	Lung	Ileocaecal tonsil	Colon	Brain	
Intranasal	4	1	30	–	–	–	–	–	–	–
	4	2	150	–	–	–	–	–	–	–
	14	3	30	10	–	–	–	–	–	–
	14	4	10	60	–	10	170	200	350	+
	14	Sentinel	–	–	–	–	–	–	–	–
Alimentary	4	1	–	–	5	–	–	–	–	–
	4	2	30	–	–	–	–	–	–	–
	14	3	20	–	–	–	–	–	–	–
	14	4	140	–	–	–	–	–	–	–
	14	Sentinel	–	–	–	10	–	–	–	–
Intranasal positive control‡	4	1	300,000	10,000	860,000	5,400	NA	NA	NA	–
	4	2	83,000	35,000	56,000	1,500	NA	NA	NA	–
	4	3	750,000	4,300	1,600,000	1,500	NA	NA	NA	–
	4	4	710,000	29,000	520,000	1,100	NA	NA	NA	–

*Shown are only tissues for which >1 animal has tested positive. GEq, genome copy equivalents per 0.1 mL calculated on the basis of qRT-PCR quantification of cycle values; HPAIV, highly pathogenic avian influenza; ID, identification; NA, not applicable; qRT-PCR, quantitative reverse transcription PCR; +, positive; –, negative. †Nasal swabs positive at day 2 postinfection for the intranasal group and day 4 postinfection for the alimentary group. ‡Four pigs, infected with the same device and dose of a swine-adapted influenza A virus strain (subtype H1avN1, clade 1C2.1) of a former study are included for comparison (*9*). Pigs were sacrificed at day 4 post infection when no seroconversion was to be expected; gastrointestinal tissues and brain were not examined.

In conclusion, only 1 of 8 pigs inoculated intranasally with HPAI virus H5N1 underwent transient, low-level infection that resulted in the presence of viral RNA in several tissue specimens and seroconversion at 14 dpi. In naturally infected wild mammals, this virus was prominently detected in the brain (*2*). Given the detection of viral RNA in the brain of 1 intranasally inoculated pig, it cannot be excluded that longer observation might have revealed continuing viral replication in the brain of this animal. Sialic acid α2,3-gal receptors dominate on porcine brain cells, which might have fostered replication of α2,3-adapted viruses, such as HPAI virus H5N1 (*10*).

Overall, we conclude that pigs are unlikely vehicles in transmitting this genotype of HPAI virus H5N1 clade 2.3.4.4b among pigs and across interfaces. However, considering the ongoing massive panzootic of this virus, a plethora of new genotypes of the circulating strain is emerging, with possibly higher permissiveness for pigs. Therefore, swine populations need to be part of HPAI virus surveillance programs, and periodic reassessment of prepandemic propensity of circulating HPAI virus H5N1 genotypes in the swine model is required.
